# Methanol-Related Fatalities in Jeddah, Saudi Arabia: A 5-Year Post-Mortem Multi-Matrix Study

**DOI:** 10.3390/toxics14040308

**Published:** 2026-04-03

**Authors:** Ahmed I. Al-Asmari, Atheer Zarnoogi, Hassan Alharbi, Ahmed Alghamdi, Faiz Alsolami, Abulnasser E. Alzahrani, Sultan A. Alahmadi, Naif H. Alotaibi, Khaled A. Alboug, Mansour A. Alzahrani, Torki A. Zughaibi

**Affiliations:** 1 Special Toxicological Analysis Section, Pathology and Laboratory Medicine Division, King Faisal Special Hospital and Research Center, P.O. Box 3354, Riyadh 11211, Saudi Arabia; 2 Faculty of Medicine, Alfaisal University, Riyadh 11533, Saudi Arabia; 3 King Fahd Medical Research Center, King Abdulaziz University, P.O. Box 80216, Jeddah 21589, Saudi Arabia; 4 Department of Medical Laboratory Sciences, Faculty of Applied Medical Sciences, King Abdulaziz University, P.O. Box 80216, Jeddah 21589, Saudi Arabia; 5 Poison Control and Forensic Chemistry Center, P.O. Box 21543, Jeddah 21176, Saudi Arabia; 6 Forensic Medical Services Center, Ministry of Health, Riyadh 12382, Saudi Arabia

**Keywords:** forensic toxicology, methanol, post-mortem, distribution, ethanol

## Abstract

Although alcohol consumption is prohibited in Saudi Arabia, methanol poisoning outbreaks continue to occur, largely through surrogate or non-beverage alcohols. To date, systematic forensic documentation of methanol-related fatalities in Saudi Arabia remains limited. This study presents a comprehensive post-mortem series of methanol-related deaths investigated in Jeddah and characterizes the demographic patterns, circumstances of death, post-mortem interval (PMI), and methanol distribution across multiple biological matrices. In total, 34 post-mortem cases with toxicologically confirmed methanol exposure were retrospectively examined. Methanol and ethanol levels in blood, urine, vitreous humor, bile, gastric contents, and selected tissues were quantified using a validated headspace gas chromatography–flame ionization detection method. Decedents were aged 18–73 years (median, 34.5 years), with a marked predominance of young-to-middle-aged men. PMI ranged 1–15 days (median, 2 days), and evidence of putrefaction was present in approximately one-third of the cases. Most deaths were classified as accidental and primarily occurred in private residences. Two distinct outbreak periods (2018 and 2022) were identified; values tended to be higher in 2018, but the differences were not statistically significant. NaF-preserved blood, urine, and vitreous humor were informative in non-decomposed cases, whereas vitreous humor and solid organs, particularly the liver and kidneys, retained quantifiable methanol in putrefied bodies. Ethanol was detected in a minority of cases and was considered a secondary or contributory finding. This study provides an important forensic baseline dataset for methanol-related fatalities in Saudi Arabia and underscores the need for continued surveillance and preventive measures.

## 1. Introduction

Methanol, also known as wood or methyl alcohol, is a common component of both household and commercial products. Unfortunately, methanol abuse can occur intentionally or unintentionally [1]. Methanol can cause severe intoxication, metabolic acidosis, and vision problems. In Saudi Arabia, where alcohol consumption is banned for religious and health reasons [2,3], reports of methanol poisoning outbreaks have occurred both before and after the COVID-19 pandemic [4,5,6,7,8,9]. Interruptions in the availability of regulated alcoholic drinks likely precipitated an increase in the consumption of unregulated substitutes tainted with methanol. Unfortunately, the danger of methanol poisoning is often underestimated because of religious, cultural, and social influences. Individuals experiencing mild symptoms might refrain from seeking medical help because of fears of legal repercussions [6,10].

Studies on methanol intoxication in Saudi Arabia have revealed significant public health concerns, particularly attributable to the illicit nature of alcohol consumption in the country [4,11]. Delayed medical intervention represented a critical factor influencing mortality rates across multiple outbreaks and case reports, and fatal cases were linked to methanol concentrations exceeding 250 mg/dL [12]. The clinical presentation of methanol poisoning consistently involved severe metabolic acidosis, high anion gap values, and neurological complications, with some patients experiencing permanent vision loss because of optic nerve damage [13]. Forensic toxicology methods, particularly headspace gas chromatography–flame ionization detector (HS–GC–FID), proved highly reliable for toxicological confirmation, whereas emergency departments often utilized blood gas analysis, which lacks forensic validation [6]. Intracerebral hemorrhage, a rare but devastating complication, was documented in a fatal case, emphasizing the need for early neuroimaging in severe poisoning incidents [14].

Although most cases of methanol poisoning are not fatal, long-term complications such as irreversible blindness, basal ganglia necrosis, and cognitive decline are common among survivors [13]. Magnetic resonance imaging has frequently detected putaminal necrosis, reinforcing methanol’s neurotoxic effects [13]. Post-mortem toxicology confirmed extreme methanol levels in fatal cases, highlighting the lethal effects of delayed intervention [11,12]. Overall, the findings emphasize the urgent need for stronger toxicology screening capabilities, standardized treatment protocols, and public health awareness campaigns to minimize fatalities and prevent long-term neurological impairment following methanol poisoning [4,6]. Expanded access to antidotes such as fomepizole and timely hemodialysis are essential to reduce mortality and limit lasting health consequences [12].

When methanol is mistakenly ingested instead of or alongside ethanol, the resulting toxicity is primarily attributable to methanol itself. Trace levels of ethanol were detected in some cases, complicating the clinical picture and potentially contributing to fatal outcomes. The systematic reporting of co-detected drugs in forensic methanol deaths remains regionally limited, underscoring the urgent need for further research to understand substance abuse patterns among individuals with methanol intoxication.

Notably, most reported cases involved surviving patients, with clinical observations often relying on blood gas analysis, which is not fully validated for methanol quantification, whereas only a few reports included comprehensive post-mortem toxicological data. This disparity highlights a critical gap in understanding the true prevalence and severity of methanol poisoning in the region. The documented post-mortem cases both confirm the existence of this significant public health problem and urgently alert authorities regarding its magnitude. Consequently, these findings underscore the need for enhanced intervention measures, including rigorous post-mortem toxicology, targeted prevention programs, and educational initiatives, to reduce the incidence of methanol poisoning in Saudi Arabia.

This study primarily aimed to document and characterize methanol-related fatalities in Jeddah, Saudi Arabia, in 2018–2022 by extensively analyzing the post-mortem distribution of methanol in various bodily fluids and tissues. Detailed toxicological analyses were performed to quantify methanol concentrations in these samples, thereby establishing methanol as the principal toxic agent. Moreover, this study sought to clarify whether intoxication occurs following methanol consumption alone or along with other toxic substances.

## 2. Materials and Methods

### 2.1. Biological Specimens and Reagents

Biological specimens, including whole blood, urine, vitreous humor, gastric contents, and bile, were collected during autopsy, when available. Peripheral whole blood (subclavian) was immediately preserved with 1–2% NaF (Sigma-Aldrich, St. Louis, MO, USA) to inhibit microbial activity and minimize post-mortem production or degradation of alcohols. No preservatives were routinely added to urine or the other matrices. In selected cases, additional non-preservative tissue specimens (liver, kidneys, brain, and stomach wall) were excised, homogenized, and processed under identical conditions.

Post-mortem specimens were collected into airtight, screw-cap containers and filled as much as practicable to minimize headspace. Samples were transported and stored refrigerated (2–8 °C) and analyzed as soon as practicable within the routine laboratory workflow; when analysis could not be performed promptly, aliquots were stored frozen (≤−20 °C) until testing. Analytical standards for methanol and ethanol (Merck, Poole, UK) were used to prepare matrix-matched calibration and quality control (QC) samples. Tert-butanol (Sigma-Aldrich, St. Louis, MO, USA; 80 mg/dL in water) was used as an internal standard.

Solid tissues collected at autopsy were handled in two ways depending on case condition and available material. When appropriate, aliquots of tissue (≤10 g) were placed with internal standard (tert-butanol, 80 mg/dL) into 20 mL glass headspace vials sealed with rubber septa and analyzed directly. In selected cases, tissues were additionally minced and homogenized with distilled water (1:2, *w*/*v*) in a Stomacher^®^ bag using a Stomacher^®^ laboratory homogenizer (Seward Ltd., Worthing, West Sussex, UK); an aliquot equivalent to 1 g tissue (or 1 mL fluid) plus internal standard was then transferred to 20 mL headspace vials for HS–GC–FID analysis.

### 2.2. Sample Preparation

For both calibrators/QC samples and case specimens, 1.0 mL of whole blood, urine, or homogenized tissue supernatant was transferred into a sealed headspace vial. Then, 200 µL of tert-butanol (80 mg/dL) was added as the internal standard, and the vials were immediately closed with airtight crimp caps to prevent evaporation loss and ensure equilibrium between the liquid and gas phases prior to injection. All samples were gently mixed and maintained under controlled conditions until headspace analysis.

### 2.3. HS–GC–FID Instrumentation and Chromatographic Conditions

Two independently validated HS–GC–FID systems were used to provide robust quantification and cross-platform confirmation of the results.

#### 2.3.1. System A

System A comprised a Clarus^®^ 580 GC system (PerkinElmer, Waltham, MA, USA) coupled to a TurboMatrix HS 40 headspace autosampler (PerkinElmer, Waltham, MA, USA). Chromatographic separation was achieved using an Elite BAC-2 capillary column (PerkinElmer, Shelton, CT, USA, 30 m length, 0.32 mm i.d., 1.2 µm film thickness), which was specifically designed for forensic alcohol analysis. The GC oven was operated isothermally at 40 °C. The injector and FID detector were maintained at 200 °C. The injector was used in split mode with a split flow of 20 mL/min. The transfer line temperature was set at 200 °C using FS Hydroguard™ tubing (0.32 mm i.d., PerkinElmer) to minimize volatile component adsorption and degradation.

Helium served as the carrier gas and was delivered at a constant flow rate. The detector gases were nitrogen (30 mL/min), air (400 mL/min), and hydrogen (35 mL/min), following the manufacturer’s recommendations for optimal FID performance. The headspace sampling conditions included a 2.5 mL gas-tight syringe, an incubation time of 5 min, and an agitator temperature of 80 °C to promote equilibrium and reproducible partitioning of methanol, ethanol, and the internal standard into the gas phase. The total analysis time was 8.5 min for each injection.

#### 2.3.2. System B 

System B consisted of a Thermo Finnigan Trace GC Ultra gas chromatograph (Thermo Fisher Scientific, Milan, Italy) equipped with a TriPlus RSH™ headspace autosampler (Thermo Fisher Scientific, Waltham, MA, USA). Separation was performed on an Elite BAC-1 capillary column (PerkinElmer, Shelton, CT, USA, 30 m length, 0.32 mm i.d., 1.8 µm film thickness), which offered complementary selectivity for light alcohols and the internal standard (IS). The oven temperature program began at 40 °C (1 min hold), followed by a ramp of 20 °C/min to 110 °C, with a final 2 min hold at 110 °C. The injector was operated at 250 °C in split mode with a split flow of 18 mL/min. The detector and transfer line temperatures were maintained at 200 °C, ensuring consistency with System A in terms of detection conditions. The carrier and detector gas types, flow rates, and total chromatographic run times were identical to those used in the PerkinElmer system.

### 2.4. Method Validation

The method for quantitative methanol and ethanol determination in post-mortem specimens was validated in accordance with ANSI/ASB Standard 036 (2019) and Standard Practices for Method Validation in Forensic Toxicology [15]. The validation design assessed linearity, limits of detection (LOD) and quantification (LOQ), accuracy (bias), precision (imprecision), dilution integrity, and overall method performance in fortified post-mortem matrices. Experiments were conducted using spiked whole blood, and urine prepared with matrix-matched calibrators and QC samples.

Calibration curves for both methanol and ethanol were prepared in whole blood and urine by spiking blank post-mortem matrices with appropriate volumes of standard solutions to yield final concentrations of 10, 25, 50, 80, 100, 200, 300, and 400 mg/dL. All calibrators were prepared in duplicate and processed identically to the case samples. Following ANSI/ASB Standard 036, which allows the use of calibrators prepared in a representative matrix for quantitation in other matrices provided that acceptable bias and precision are demonstrated, these whole blood and urine calibration curves were applied to all post-mortem specimens, including the vitreous humor, bile, and tissue homogenates. Dedicated matrix-specific calibration curves were not generated for individual tissues, as tissues are infrequently submitted for alcohol analysis in routine casework, and validation experiments demonstrated that quantitation using whole blood/urine calibrators met the predefined accuracy and precision criteria across all matrices.

Aqueous QC standards (10, 25, 80, and 300 mg/dL) were included in each analytical batch to monitor the accuracy and reproducibility across the calibration range and over time. These QC samples were treated as unknowns and subjected to the complete HS–GC–FID procedure.

#### 2.4.1. Linearity and Analytical Sensitivity

Linearity was evaluated for both methanol and ethanol over a range of 10–400 mg/dL using calibrators at 10, 25, 50, 80, 100, 200, 300, and 400 mg/dL in post-mortem whole blood and urine samples. The calibration curves generated by both HS–GC–FID systems consistently exhibited excellent correlation, with a coefficient of determination > 0.999 for both analytes across all validation runs. LOD was 5.0 mg/dL for ethanol and 2.5 mg/dL for methanol, whereas the LOQ was 10 mg/dL for ethanol and 5 mg/dL for methanol. The upper LOQ was validated at 400 mg/dL for both analytes.

#### 2.4.2. Accuracy and Precision of LOQ

At the LOQ, both analytes demonstrated comparable performance. Bias (accuracy) ranged from −2% to +4% (median ≈ 2%, SD ≈ 3%) for ethanol and methanol. The within-run precision at LOQ was 6–12% CV (median ≈ 11%, SD ≈ 3%), and the between-run precision ranged from 7–10% CV across both instruments and matrices. These results satisfy the commonly applied forensic toxicology acceptance criteria for quantitative methods at the LOQ.

#### 2.4.3. Matrix Performance in Post-Mortem Whole Blood Samples

In NaF-preserved post-mortem whole blood, ethanol exhibited bias between −3% and +1% (median = −2%, SD ≈ 1%) at QC concentrations of 10, 25, 80, and 300 mg/dL. The within-run precision ranged 2–8% CV (median = 3%, SD ≈ 2%), and the between-run precision ranged 1–5% CV (median = 3%, SD ≈ 2%). Methanol exhibited a similarly robust performance in blood, with a bias within the range of approximately −3% to +3% and imprecision ≤ 8% CV across the same QC levels, demonstrating stable quantitative behavior over time for both HS–GC–FID systems.

#### 2.4.4. Matrix Performance in Post-Mortem Urine Samples

In post-mortem urine, the accuracy for ethanol ranged from −1% to +8% (median = −2%, SD ≈ 3%) across QCs of 10, 25, 80, and 300 mg/dL. The within-run precision was 1–3% CV (median = 3%, SD ≈ 1%), and the between-run precision ranged 1–4% CV (median = 3%, SD ≈ 1%). Comparable performance was observed for, with bias ranging from approximately −3% to +8%, within-run imprecision of ≤3% CV, and between-run imprecision of ≤4% CV across the same QC levels. For both analytes, the accuracy and precision in urine were within the commonly accepted forensic validation limits for the quantitative analysis of volatiles in urine.

#### 2.4.5. Dilution Integrity

Dilution integrity was evaluated at both low and high dilution factors. A 1:10 dilution of a 200 mg/dL control sample (nominal 20 mg/dL) produced measured concentrations of 20–21 mg/dL (median ≈ 21 mg/dL, SD ≈ 1 mg/dL). A 1:100 dilution of a 20,000 mg/dL control sample (nominal 200 mg/dL) yielded values of 198–202 mg/dL (median ≈ 197–200 mg/dL, SD ≈ 2 mg/dL). Both ethanol and methanol remained within approximately ±10% of their nominal values at each dilution.

### 2.5. Case Samples

For post-mortem medicolegal cases recorded in the forensic toxicology databases of the Jeddah Poison Control and Forensic Chemistry Center, methanol concentrations and related case information (history, mode of death, and manner of death) were retrieved and summarized following ethical approval (Research Code: 00842; Ethical Approval Committee, Jeddah Health Affairs, Ministry of Health, Jeddah, Saudi Arabia; Approval No.: A00508). The study included cases received between 1 January 2018, and 31 December 2022, in which methanol intoxication was suspected as a contributing factor to death in the Jeddah region.

Routine post-mortem sampling included peripheral whole blood preserved with NaF and urine without preservatives, with additional matrices collected in selected cases, including liver, kidney, brain, stomach wall, spleen, skeletal muscle, bile, and vitreous humor samples. Specimens were obtained at autopsy or, when applicable, during hospital admission prior to death.

For each case, demographic and contextual information was recorded, including age, sex, body weight, and height, post-mortem interval (PMI), place of death (home, hospital, outdoor environment, workplace, vehicle, or hotel), and relevant medical and toxicological histories. Several cases involved co-exposure to other substances (e.g., ethanol, amphetamines, Δ^9^-THC, benzodiazepines, and lidocaine), whereas others involved advanced putrefaction or unusual exposure scenarios, such as ingestion of battery water or cologne.

All specimens were subjected to a comprehensive toxicological workup, comprising immunoassay, quantitative alcohol analysis, carbon monoxide determination, general unknown screening, and confirmatory testing using HS–GC–FID and GC–mass spectrometry, as appropriate.

### 2.6. Statistical Analysis and Figure Preparation

Descriptive statistics were used to encapsulate demographic details, the interval between death and examination, the cause and place of death, the state of decomposition, and methanol levels across different matrices. Continuous variables are presented as medians with ranges or interquartile ranges, whereas categorical variables are presented as counts and percentages. Because methanol concentrations were non-normally distributed and sample sizes were limited for several matrices, non-parametric statistical methods were applied throughout the study. Group comparisons were performed using the Mann–Whitney U test, with effect sizes expressed as rank-biserial correlation coefficients, and the Holm adjustment was applied where appropriate. Spearman’s rank correlation coefficient was utilized to evaluate the relationships between methanol levels detected in various matrices. All statistical analyses were conducted using IBM SPSS Statistics for Windows, Version 29.0 (IBM Corp., Armonk, NY, USA). Data handling, stratification, and figure preparation were conducted using Microsoft Excel for Microsoft 365 (Microsoft Corporation, Redmond, WA, USA), with all graphs generated directly from the final dataset and formatted according to forensic toxicology publication standards.

## 3. Results

### 3.1. Demographic Profile

**Figure 1:**
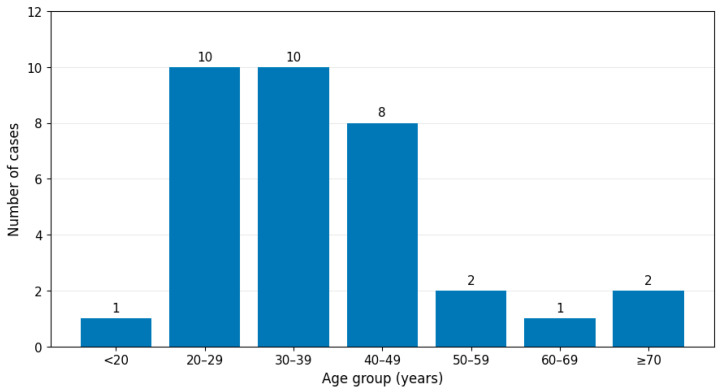
Age distribution of methanol-related fatalities (*n* = 34). Most decedents (82.4%) were young-to-middle-aged adults (20–49 years).

The individuals ranged in age from 18 to 73 years (mean, 36.9 ± 13.8 years; median, 34.5 years). A clear predominance of young-to-middle-aged adults was observed, with individuals aged 20–49 years accounting for 28 of the 34 cases (82.4%; Figure 1). There was a marked male predominance, with males representing 28 cases (82.4%), consistent with the patterns of exposure to surrogate or non-beverage alcohol. No putrefaction was documented in 23/34 cases (67.6%). Conversely, some putrefaction was observed in seven cases (20.6%), and advanced decomposition was noted in four cases (11.8%), which generally corresponded to longer PMIs.

**Figure 2:**
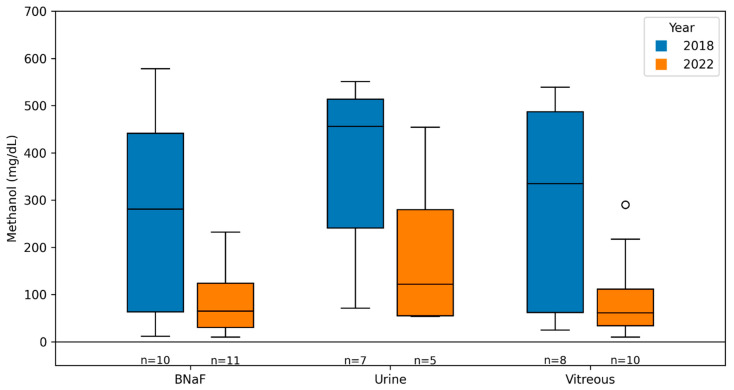
Methanol concentrations in key biofluids during outbreak years (2018 vs. 2022). Boxplots show methanol concentrations (mg/dL) in NaF-preserved blood (BNaF), urine, and vitreous humor. Boxes represent the median and interquartile range; whiskers indicate dispersion and points indicate outliers. Sample sizes: BNaF (2018 *n* = 10; 2022 *n* = 11), urine (2018 *n* = 7; 2022 *n* = 5), vitreous (2018 *n* = 8; 2022 *n* = 10).

Multi-matrix sampling was used for subsequent analyses. NaF-preserved blood, vitreous humor, urine, and peripheral blood were available in 26 (76.5%), 22 (64.7%), 16 (47.1%), and 12 cases (35.3%), respectively. Solid tissues, including the liver (9/34, 26.5%) and kidneys (8/34, 23.5%), were also frequently retained, supporting the need for a detailed assessment of methanol distribution across fluids and tissues. A summary of the demographic characteristics, death circumstances, PMI, putrefaction status, and specimen availability is provided in Table 1. Methanol concentrations measured during the two outbreak years across major biofluids are compared in Table 2 and Figure 2, providing a temporal context for the cohort.

### 3.2. Age Groups and Analyte Concentrations

Across the cohort, methanol concentrations in biofluids, including NaF-preserved blood, peripheral blood, urine, vitreous humor, gastric contents, and bile, ranged 10–610 mg/dL. When pooled across matrices, the concentrations exhibited marked dispersion (median ≈ 130 mg/dL; mean ≈ 200 mg/dL; SD ≈ 182 mg/dL; *n* = 86 measurements). In solid tissues (liver, kidneys, stomach wall, bladder tissues, intestine, brain, spleen, and skeletal muscle), methanol concentrations ranged 0.016–202 mg/g (median ≈ 1.1 mg/g; mean ≈ 17.9 mg/g; SD ≈ 44.8 mg/g; *n* = 27), confirming extensive post-mortem distribution into both parenchymal organs and hollow viscera. As shown in Table 3, only specimens suitable for quantitative analysis were included. Table 1 summarizes matrices collected/available at autopsy; some available specimens were excluded from quantification due to insufficient volume or compromised condition.

**Figure 3:**
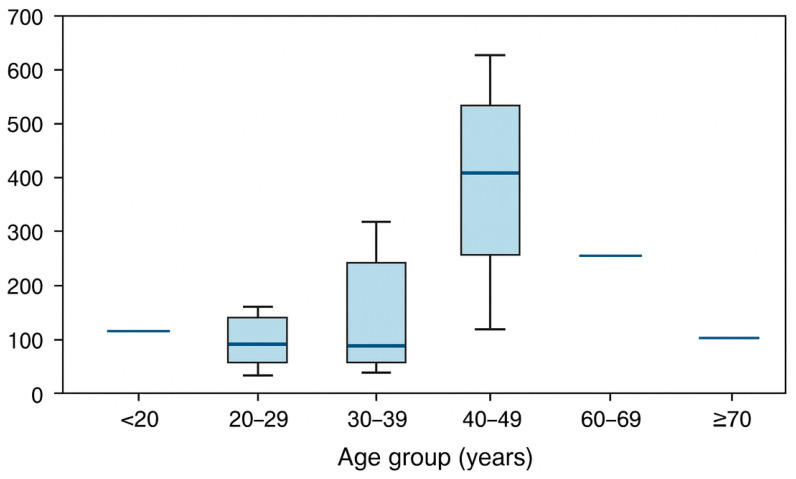
Methanol concentrations in NaF-preserved blood (mg/dL) by age group among cases with available samples (*n* = 26). Boxplots show the median and interquartile range, with whiskers indicating dispersion and points indicating outliers. High concentrations were observed across multiple adult age strata; however, some age groups had small sample sizes, and results are interpreted descriptively.

Matrix-specific evaluation demonstrated distinct concentration profiles for each matrix. The methanol concentration in NaF-preserved blood (*n* = 26) ranged 10–578 mg/dL (median, 101 mg/dL), whereas that in peripheral blood (*n* = 12) ranged 11–610 mg/dL (median, 244 mg/dL). Urine (*n* = 15) frequently exhibited high concentrations (54–551 mg/dL; median, 310 mg/dL), consistent with renal elimination, whereas that in vitreous humor (*n* = 22) ranged 10–539 mg/dL (median, 90 mg/dL), often retaining measurable methanol content even in cases with post-mortem changes. The liver (*n* = 8) and kidneys (*n* = 6) typically exhibited lower but clearly toxic concentrations in the low mg/g range (median ≈ 1.7 and 2.1 mg/g, respectively), with occasionally markedly elevated values (Table 3). Methanol was detected in NaF-preserved blood all adult age strata. Lethal BNaF concentrations (>250 mg/dL) were observed in young adults (20–29 years), middle-aged adults (30–49 years), and older individuals, indicating that fatal intoxication was not confined to a specific age group. The age-stratified distributions of NaF-preserved blood methanol are presented in Figure 3. Spearman’s rank correlation demonstrated a modest positive association between age and NaF-preserved blood concentrations (ρ ≈ 0.44), with substantial overlap between age groups, underscoring marked interindividual variability.

Ethanol was detected in only a small subset of cases and matrices tested. In these mixed-exposure cases, methanol concentrations remained within toxic or lethal ranges, and methanol was considered the dominant contributor to death, with ethanol regarded as a secondary or potentially contributory finding.

### 3.3. PMI, Putrefaction, and Methanol Concentrations

**Figure 4:**
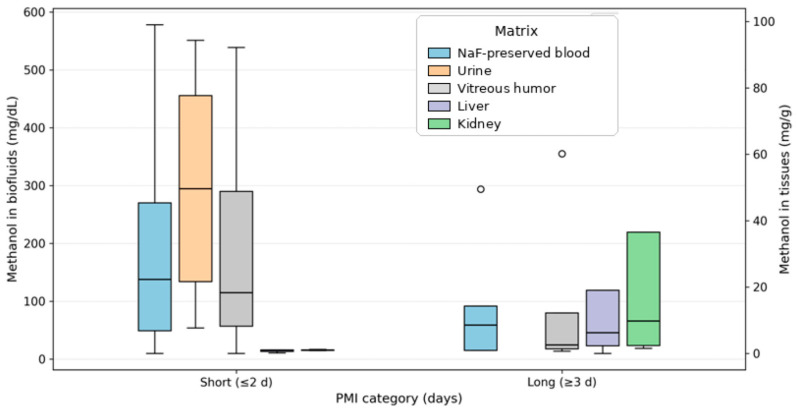
Methanol concentrations by PMI category across key matrices. Boxplots compare short PMI (≤2 days) vs. longer PMI (≥3 days) for NaF-preserved blood, urine, vitreous humor (mg/dL) and liver/kidney (mg/g); biofluids and tissues are shown on separate y-axes. In each boxplot, the central line represents the median, the box spans the interquartile range (IQR), whiskers extend to 1.5× IQR, and individual dots represent observed data points/outliers.

In this series, PMI ranged 1–15 days, with most examinations performed within the first 10 days after death (median, 2 days; mean, 2.65 ± 2.84 days). The interpretation of methanol concentrations in relation to PMI and the recorded degree of putrefaction revealed systematic differences in matrix reliability. In cases without putrefaction, the NaF-preserved blood methanol concentrations ranged 10–578 mg/dL (mean 142.8 mg/dL; *n* = 21). Conversely, cases with putrefactive changes exhibited higher average NaF-preserved blood concentrations (mean, 283.8 mg/dL; *n* = 5), consistent with greater dispersion and post-mortem effects rather than a uniform directional shift. This pattern resulted in a modest positive association between the putrefaction status and NaF-preserved blood methanol concentration (Spearman ρ ≈ 0.34), as summarized in Table 4. To further visualize PMI effects (Figure 4), we stratified cases into short PMI (≤2 days) and longer PMI (≥3 days) categories and compared methanol concentrations across key matrices (NaF-preserved blood, urine, vitreous humor, liver, and kidney), highlighting increased dispersion in blood with longer PMI and the continued interpretive utility of vitreous and tissues.

A comparable trend was observed in solid tissues. The average liver methanol concentration in non-putrefied cases was 5.1 mg/g (*n* = 4), versus 27.2 mg/g in putrefied cases (*n* = 4), yielding a similar positive association with putrefaction (ρ ≈ 0.35). Contrarily, the methanol concentrations in vitreous humor displayed minimal dependence on putrefaction, with nearly identical mean values in non-putrefied and putrefied cases (179.6 mg/dL vs. 181.4 mg/dL; ρ ≈ 0.00; *n* = 22). Urine concentrations were likewise stable across putrefaction categories (305.4 mg/dL vs. 300.8 mg/dL; ρ ≈ −0.01; *n* = 15).

These matrix-specific patterns, as illustrated in Figure 4, demonstrate the increased variability and reduced interpretive reliability of blood in decomposed cases, in contrast to the relative stability of the vitreous humor and solid organs. This effect was exemplified by case 16, who had a prolonged PMI of 15 days and advanced putrefaction. In this patient, blood was analytically compromised, whereas vitreous humor and tissue samples retained robust methanol concentrations, supporting the diagnosis of fatal intoxication.

These findings indicate that although NaF-preserved blood remains informative in non-decomposed cases, vitreous humor and solid organs provide more reliable evidence of methanol exposure as PMI increases and putrefaction progresses, reinforcing the necessity of a multi-matrix approach across the full PMI spectrum.

### 3.4. Mode of Death

**Figure 5:**
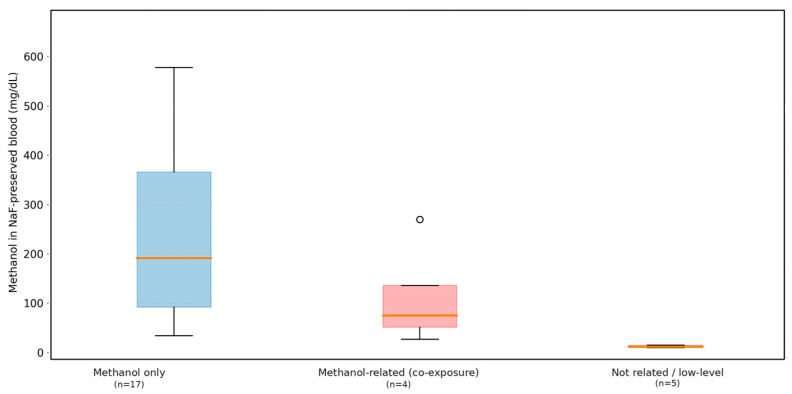
Methanol concentrations in NaF-preserved blood (mg/dL) by toxicological classification (*n* = 26). Boxplots show the median and interquartile range; whiskers indicate dispersion and points indicate outliers.

The predominant cause of death, as determined by quantitative toxicological findings and supported, as available, by clinical and investigative information, was acute methanol poisoning. In cases with documented pre-mortem medical data, features consistent with methanol toxicity were reported, including severe metabolic acidosis, visual disturbances, neurological impairment (seizures and coma), and rapid clinical deterioration despite medical intervention. As summarized in Table 5 and Figure 5, substantial overlap of NaF-preserved blood methanol concentrations was observed across the different modes of death, reflecting differences in survival time, the extent of metabolic conversion to formate, and the presence or absence of medical treatment, rather than differences in exposure magnitude alone.

A subset of cases involved co-exposure to ethanol and other psychoactive substances. In these cases, methanol concentrations remained within the established toxic or lethal ranges. As illustrated in Table 5 and Figure 5, methanol was considered the primary toxic agent, whereas ethanol and other substances were interpreted as contributory or complicating findings rather than the principal causes of death. Thus, the toxicological concentration alone did not distinguish between patients’ clinical courses or investigative classifications of death, underscoring the necessity of integrating quantitative toxicology with clinical history and scene information when determining the mode of death in methanol intoxication.

### 3.5. Manner of Death

**Figure 6:**
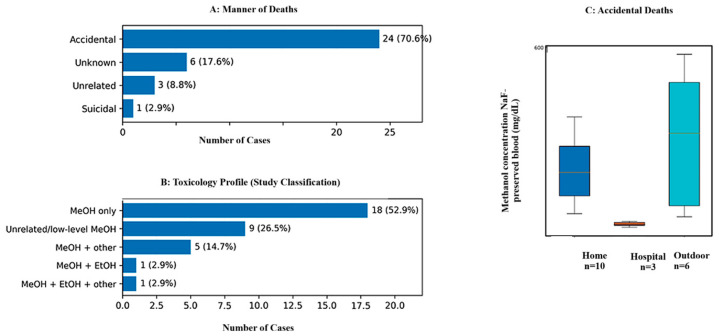
Integrated overview of manner of death, toxicological classification, and methanol concentrations by location. (**A**) Distribution of manner of death (*n*, %). (**B**) Study of toxicological classification (*n*, %). (**C**) Methanol concentrations in accidental deaths stratified by location (home, hospital, outdoor); boxplots show median and IQR with whiskers indicating dispersion and points indicating outliers.

Accidental deaths accounted for 24 of 34 events (70.6%), reflecting unintentional ingestion of methanol-containing surrogate or adulterated products by the victims. A further six cases (17.6%) were classified as undetermined, largely because of limited circumstantial information, advanced decomposition, or uncertainty regarding intent at the scene. Only one case (2.9%) was classified as suicide, involving deliberate exposure, whereas three cases (8.8%) were considered unrelated to methanol intoxication following a full medicolegal evaluation. Methanol concentrations displayed substantial overlap between accidental and undetermined cases. As presented in Table 6 and Figure 6, methanol concentrations in NaF-preserved blood and other matrices could not permit reliable discrimination of intent based on toxicological data alone. Cases classified as unrelated to methanol intoxication generally featured lower or inconsistent methanol findings across matrices, supporting their exclusion from the methanol-causal interpretation.

These distributions demonstrate that toxicological severity alone is insufficient to infer the manner of death, particularly in cases with limited investigative data or post-mortem changes.

### 3.6. Location of Deaths and Putrefaction

Most deaths in private residences or shared accommodations (18/34, 52.9%), consistent with the ingestion of surrogate alcohols or improperly stored methanol-containing products (Table 1). Seven deaths (20.6%) occurred in hospitals, representing individuals who survived the initial intoxication but later died despite medical intervention. Because some decedents received hospital management prior to death (e.g., ethanol therapy and/or intravenous fluids), measured methanol/ethanol concentrations in these cases may reflect treatment-related effects (dilution and/or altered kinetics) and are interpreted accordingly. The remaining deaths were discovered in outdoor or public settings (6/34, 17.6%), including workplaces, vehicles, and hotels. Evidence of putrefaction was documented in 11 cases (32.4%). Putrefaction was uncommon in hospital deaths, but it occurred more frequently in deaths discovered at home or in outdoor environments, particularly when PMI exceeded 5 days. In these cases, peripheral and NaF-preserved blood samples were often hemolyzed or otherwise analytically compromised, limiting their reliability for methanol quantification.

**Figure 7:**
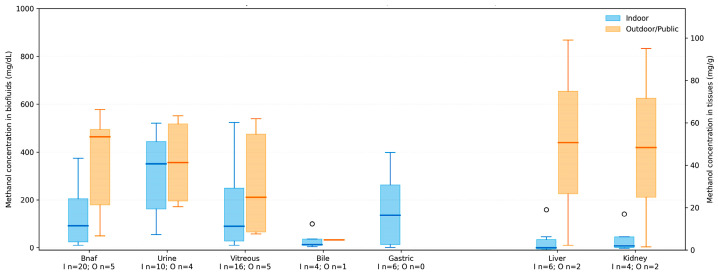
Methanol concentrations by biological matrix and discovery setting. Boxplots compare indoor and outdoor/public cases across major biofluids and tissues; blue denotes indoor and yellow denotes outdoor/public. Sample sizes for each matrix and setting are indicated.

**Figure 8:**
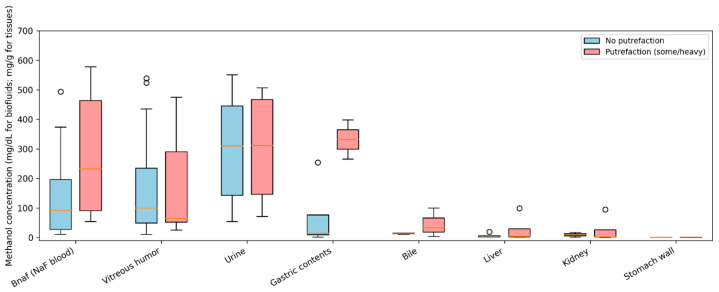
Methanol concentrations in biofluids and tissues by putrefaction status. Blue boxplots represent cases without putrefaction; red boxplots represent cases with some/heavy putrefaction. Boxplots show the median and interquartile range; whiskers indicate dispersion and points indicate outliers. Vitreous humor and solid tissues frequently retained quantifiable methanol in decomposed cases.

By contrast, vitreous humor, bile, and solid organs, particularly the liver and kidneys, frequently retained measurable and internally consistent methanol concentrations, even in moderately to heavily decomposed cases. The distribution of methanol concentrations by discovery location and degree of putrefaction is illustrated in Figure 7 and Figure 8. The data demonstrate increased variability in blood measurements in decomposed cases and greater stability in alternative matrices. These findings illustrate that the location of death influences post-mortem conditions and matrix suitability for toxicological interpretation. In cases involving delayed discovery or environmental exposure, interpretation based on multiple matrices provides more reliable evidence of methanol exposure than reliance on blood alone. Importantly, concentrations measured in solid tissues (mg/g) and body fluids (mg/dL) are expressed in different units and reflect distinct biological compartments. Therefore, these values should not be interpreted as directly comparable dose-equivalent measures across matrices without normalization or partitioning considerations. In this study, the multi-matrix approach is used primarily to support confirmation of exposure, strengthen interpretation in decomposed cases, and assess matrix suitability, rather than to infer quantitative cross-matrix dose relationships.

### 3.7. Seasonal Distribution

**Figure 9:**
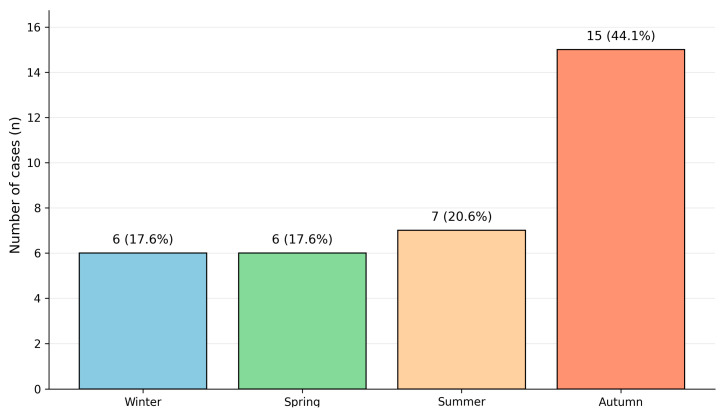
Seasonal distribution of methanol-related fatalities using a four-season model (winter, spring, summer, and autumn) for cases with available information (*n* = 34).

As summarized in Table 7 and Figure 9, methanol-related mortality most frequently occurred between November and February. These events were predominantly associated with indoor settings, including private residences and shared accommodation. Fewer deaths occurred during the summer (June–August). Although less frequent, summer events were more often characterized by delayed discovery and longer PMIs, frequently accompanied by moderate-to-severe putrefaction, consistent with higher ambient temperatures. In these cases, blood samples were more commonly compromised, and methanol interpretation relied more heavily on vitreous humor and solid tissue concentrations. Therefore, seasonal differences were reflected in case frequency as well as post-mortem conditions and matrix availability.

### 3.8. Multiple Matrices and Methanol Concentrations

In cases with short PMIs and no evidence of decomposition, methanol concentrations in NaF-preserved blood, urine, vitreous humor, and solid tissues were generally concordant, reflecting acute systemic exposure to methanol. In these cases, methanol concentrations in blood and vitreous humor were moderately correlated (ρ ≈ 0.62), indicating that methanol content in vitreous humor closely reflected circulating levels when post-mortem changes were minimal. In cases with prolonged PMIs or putrefaction, methanol concentrations in blood displayed increased variability, whereas those in alternative matrices were more consistent. Strong correlations were observed between solid organs, particularly the liver and kidneys (ρ ≈ 0.78), and between vitreous humor and liver (ρ ≈ 0.71), supporting the relative stability of these matrices in decomposed bodies. Urine methanol concentrations also remained quantifiable across a wide range of post-mortem conditions and exhibited limited sensitivity to decomposition.

**Figure 10:**
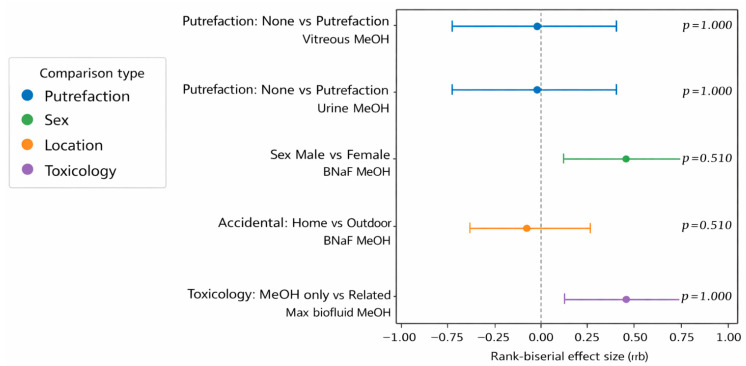
Forest plot of rank-biserial effect sizes (*r_rβ_*) from Mann–Whitney U tests comparing methanol concentrations by putrefaction status, sex, location, and toxicological classification. Points represent effect sizes and horizontal bars show 95% confidence intervals; the dashed line indicates no effect (*r_rβ_* = 0). Holm-adjusted *p*-values are shown.

Pairwise non-parametric comparisons using the Mann–Whitney U test revealed no significant differences in methanol concentrations across key categorical variables, including sex, discovery location of accidental deaths, putrefaction status, and toxicological grouping. Methanol concentrations in vitreous humor and urine did not differ significantly between cases with putrefaction, whereas NaF-preserved blood displayed greater dispersion in decomposed cases without a statistically significant shift in the central tendency. The median methanol concentration was higher in methanol-only cases than in methanol-related cases, although this difference did not reach statistical significance within the sample sizes available. These matrix-specific patterns and correlation structures are illustrated in Figure 10 and summarized in Table 8 and Table 9, demonstrating that the interpretation of methanol exposure benefited from the integration of multiple fluids and tissues, particularly in cases in which blood integrity was compromised.

## 4. Discussion 

This study conducted a detailed forensic toxicology analysis of methanol-related fatalities in Jeddah, Saudi Arabia over a 5-year period, combining a formally validated HS–GC–FID method with multi-matrix post-mortem analysis and comprehensive case metadata. Contradicting most prior Saudi reports [4,6,10,12], which largely comprised small clinical series, outbreak descriptions, or isolated case reports, this study focused exclusively on fatal cases, and methanol content was quantified in a broad range of biofluids and solid tissues. When considered alongside previous national studies, the present series fills a clear gap by offering systematically generated concentration data interpreted within a consistent analytical and forensic framework.

Between 2018 and 2022, 34 methanol-related fatalities were identified, but their temporal distribution was clearly clustered. In total, 13 and 12 deaths occurred in 2018 and 2022, respectively, accounting for the vast majority of cases. Thus, both 2018 and 2022 featured an annual case count approximately 4-fold higher than those in the intervening years, supporting the interpretation of these 2 years as distinct methanol outbreaks superimposed on a lower endemic background.

The 2018 cluster occurred entirely in the pre-COVID-19 period, and it likely reflects “classic” surrogate alcohol consumption in an environment in which legal ethanol beverages are unavailable and methanol-containing products (e.g., industrial solvents, counterfeit spirits) are used as substitutes. By contrast, the 2022 cluster arose in the late/post-COVID phase, when social and economic activities, travel, and informal gatherings in Jeddah were largely resuming after the initial pandemic restrictions. The early COVID-19 period, particularly in 2020, was marked by large methanol outbreaks, with some countries reporting hundreds to thousands of poisonings linked to illicit alcohol, methanol-containing hand sanitizers, and misconceptions about alcohol as protection against SARS-CoV-2. In several published series and scoping reviews, these pandemic-associated outbreaks caused sharp, short-lived spikes in methanol cases, often limited to a few weeks or months. The absence of a peak in 2020–2021, despite global concerns about sanitizer-related poisonings and huge methanol outbreaks reported in other countries, suggests that the local pattern did not simply follow the international trend of early pandemic methanol crises. Instead, the data indicate a more complex trajectory: a pronounced outbreak before COVID-19 (2018), a relative trough during the first years of the pandemic (2019–2021), and a second outbreak after restrictions eased (2022).

The local pattern clearly differed from the classic “COVID methanol surge.” In Iran, Shokoohi et al. [16] described a true syndemic, with approximately 5000 methanol poisonings and ≥500 deaths between February and April 2020, driven by misinformation that drinking alcohol could prevent COVID-19 and reliance on bootleg spirits in a prohibition context. A systematic scoping review by Mousavi-Roknabadi et al. [9] confirmed this by summarizing ≥5876 hospitalizations and approximately 800 deaths in early 2020 in Iran, highlighting modifiable drivers such as rumors, weak risk communication, and poor control of informal alcohol markets. In the United States, Holzman et al. [17] reported a different COVID-linked mechanism, with methanol-contaminated hand sanitizers leading to a 124% increase in sanitizer exposure and five fatal cases in Arizona in 2020. However, we observed no early-2020 spike in Jeddah; instead, methanol-involved deaths clustered in 2018 and 2022, suggesting that COVID-19 functioned mainly as an indirect modifier (reducing exposures during strict restrictions and permitting a rebound once social and economic activity resumed) rather than as the direct trigger of a single, large early pandemic mass outbreak.

Compared with earlier Saudi literature, the current cohort is larger, analytically more robust, and more comprehensive in its sampling strategy. Previous reports from different regions of the Kingdom described small numbers of cases or larger numbers of survivors presenting to emergency departments with metabolic acidosis and visual symptoms, often relying primarily on anion and osmolal gaps as indirect markers of toxic alcohol exposure. Contrarily, few publications included quantitative methanol measurements. These measurements were typically limited to blood, and they were limited by extremely small sample sizes.

Compared with autopsy-based series from neighboring and regional countries, such as the large Ankara study in Turkey [1] and outbreak-focused reports from Uttarakhand (India) [18] and Kuala Lumpur (Malaysia) [19], the current work is distinctive in scope and structure. The Ankara study pooled ethanol- and methanol-related deaths over an 11-year interval, whereas the Indian and Malaysian studies both concentrated on a single, short-lived hooch episode. By contrast, the present study was restricted to methanol, two pronounced fatality peaks were documented within a 5-year period, with additional sporadic cases in the intervening years. Demographically, all four series exhibit a predominance of young-to-middle-aged men, but the Jeddah cohort covered a wider age span from childhood to old age and included female decedents. This research uniquely spans both the pre- and post-COVID years, demonstrating that methanol deaths re-emerged after the relaxation of pandemic restrictions rather than peaking during early 2020. On this basis, the current study can be considered the largest methanol-specific post-mortem series over a 5-year interval in the region despite being conducted over a shorter timeframe than the Turkish study.

From an analytical and interpretive standpoint, the Jeddah series is also more detailed than comparable studies [1,18,19]. Although the Ankara, Uttarakhand, and Kuala Lumpur reports relied mainly on blood methanol concentrations with limited examination of other matrices, the present study employed a formally validated HS–GC–FID method on two systems and applied it systematically to multiple post-mortem matrices, quantifying methanol in biofluids and solid tissues. This design, applied to both fresh and decomposed bodies, found that vitreous humor, bile, liver, and kidney samples frequently yield more stable and informative methanol results than degraded peripheral blood at longer PMIs, an aspect only briefly mentioned or not explored in the other series. Although all four studies reported blood methanol concentrations within broadly comparable lethal ranges and consistently identified methanol as the primary toxic agent, the Jeddah dataset uniquely couples validated methodology with detailed multi-matrix distribution data, providing a more robust framework for interpreting methanol in complex post-mortem casework.

The methanol concentrations observed in this series spanned a wide range in both fluids and tissues. Importantly, the recorded range overlaps with and also extends beyond the values reported in previous fatal cases in Saudi Arabia. Earlier reports often described lethal outcomes at blood methanol concentrations lower than 300 mg/dL, whereas our series documented deaths at both comparable and substantially higher levels. This reinforces the principle that no single “lethal threshold” can be rigidly applied in forensic interpretation, as death can occur across a broad concentration spectrum influenced by the dose, rate of ingestion, survival time, availability and timing of treatment, and individual susceptibility. The inclusion of tissue data further supports the interpretation of systemic exposure, particularly in cases in which fluid specimens are compromised.

The demographic and circumstantial patterns observed in this study were broadly consistent with previous national and regional observations, but they were more clearly quantified. The observed predominance of fatality among younger males likely reflects a combination of social and occupational factors, including the clandestine consumption of surrogate alcohols among working-age men and easier access to industrial methanol-containing products in male-dominated work environments. Simultaneously, the presence of both extremely young and older decedents illustrates that vulnerability is not confined to a single age group. The variety of case locations underscores the diversity of contexts in which methanol exposure can occur. Unusual sources, such as battery water and cologne, highlight the continued availability of methanol in non-beverage products that might be repurposed or misused.

An important contribution of this study is its demonstration of the influence of PMI decomposition on methanol interpretation and the utility of a multi-matrix strategy in addressing these challenges. In cases involving short PMIs and minimal decomposition, peripheral blood and urine usually contain high methanol concentrations. However, once PMI exceeds several days, particularly in a hot climate, blood is frequently hemolyzed, discolored, and markedly affected by putrefactive changes [20,21]. In such circumstances, reliance on blood alone would make interpretation difficult and, in some instances, unreliable. The routine analysis of vitreous humor, bile, and key solid organs in this series illustrated that these matrices often retain diagnostically useful methanol concentrations when blood is compromised. In particular, vitreous humor served as a relatively stable compartment for methanol, whereas liver and kidney concentrations provided strong evidence of systemic exposure, even when peripheral blood values were low or ambiguous. These findings provide empirical support, in a sizable cohort, to the recommendation that suspected methanol deaths, especially in warm climates and with delayed discovery, should always be investigated using a multi-matrix approach rather than limiting analysis to blood. Importantly, concentrations measured in solid tissues (mg/g) and body fluids (mg/dL) are expressed in different units and reflect distinct biological compartments. Therefore, these values should not be interpreted as directly comparable dose-equivalent measures across matrices without normalization or partitioning considerations. 

Consistent with a multi-matrix approach, prior studies have shown that methanol can be quantified across alternative post-mortem matrices, including vitreous humor, urine, bile, gastric contents, and tissues. In a homicidal pediatric fatality, methanol was detected in venous blood (231 mg/dL), urine (306 mg/dL), vitreous humor (276 mg/dL), bile (252 mg/dL), and gastric contents (256 mg/dL), with additional tissue involvement (liver 1.26 mg/g), supporting broad distribution beyond blood [22]. A recent outbreak autopsy series similarly reported wide multi-matrix distribution with median methanol concentrations of 261 mg/dL in blood, 330 mg/dL in urine, 321 mg/dL in vitreous humor, and 281 mg/dL in bile, alongside quantifiable tissue concentrations, reinforcing the corroborative value of alternative matrices in post-mortem investigations [23]. A classic post-mortem distribution study reported serum methanol of 583 mg/dL on admission and post-mortem levels of 142 mg/dL in blood, 173 mg/dL in vitreous humor, and 175 mg/dL in bile, with tissue concentrations ranging from 0.93 to 1.59 mg/g and 73 mg total methanol in gastric contents, further illustrating that vitreous, bile, gastric contents, and tissues can remain informative alongside blood [24]. In our Jeddah series, urine (median 310 mg/dL; range 54–551) and vitreous humor (median 90 mg/dL; range 10–539) showed overlapping ranges with these reports, and vitreous humor exhibited minimal dependence on putrefaction (mean 179.6 vs. 181.4 mg/dL), supporting its interpretive stability when blood is compromised; however, tissue (mg/g) and fluid (mg/dL) values are used as complementary/confirmatory evidence rather than dose-equivalent measures.

Co-exposure was common in this cohort, adding another layer of interpretive complexity. Ethanol was detected in a subset of cases, sometimes at moderate or high concentrations, and other drugs, such as amphetamines, benzodiazepines, and cannabinoids, were also detected. These patterns mirror the clinical and outbreak literature, indicating that methanol intoxication often occurs in the context of polysubstance use. From a mechanistic perspective, ethanol can transiently slow methanol metabolism by competing for alcohol dehydrogenase, potentially modifying the timing of symptom onset and progression. However, in the present series, methanol concentrations were generally sufficient to explain fatality, and methanol was considered the primary toxicant in all cases in which it was detected. Co-ingestants were therefore considered modifying or contributing factors influencing clinical presentation and possibly survival time, but they did not replace methanol as the central driver of severe metabolic acidosis and death.

Seasonal and environmental factors were also relevant in this cohort. Methanol-related deaths were recorded throughout the year, but there is an apparent clustering in the cooler months, when people are more likely to remain indoors and share surrogate alcohols or other methanol-containing liquids. Specimens tended to be better preserved during cooler months, and the combination of blood and other fluids was usually sufficient for confident interpretation. Conversely, summer cases were less frequent but more likely to involve outdoor discovery and significant delays before autopsy, with advanced decomposition being evident at the time of examination. Under these conditions, the interpretive value of blood was markedly reduced, and vitreous humor and organ tissues became central to reconstructing the exposure. This seasonal pattern illustrates how climate and PMI interact to shape the available toxicological evidence and suggests that both forensic strategies and public health messaging must be tailored to local environmental conditions and seasonal risk profiles.

From an analytical standpoint, the strength of this study lies in its use of a formally validated HS–GC–FID method for both methanol and ethanol using two independent systems. Linearity over 10–400 mg/dL, low LODs and LOQs, acceptable bias and imprecision at all QC levels, and satisfactory dilution integrity collectively support the reliability of the quantitative data generated from the casework. The observed comparable performance across both platforms further reduces the likelihood that the observed interindividual variation is an artifact of instrumentation or calibration. This level of validation was not consistently documented in previous Saudi methanol studies, and thus, it represents a significant methodological advance in the local forensic toxicology field.

This study was not without limitations. Specifically, this retrospective research was confined to a single forensic center; therefore, it did not capture all methanol-related deaths that might have occurred in the region, particularly those not subjected to medicolegal investigation. Clinical information was incomplete in some cases, which limited the ability to correlate concentration, effect relationships, and treatment factors such as time to presentation or use of antidotes and hemodialysis. Additionally, sampling and pre-analytical handling were heterogeneous across this retrospective cohort. The available matrix set differed between cases, and only blood was routinely preserved with NaF, whereas urine and other matrices were not routinely preserved. These factors may introduce pre-analytical variability and limit strict within-case quantitative comparability between matrices, particularly in cases with prolonged PMI and/or decomposition. As this was a retrospective study based on routine casework, exact collection-to-analysis intervals were not available for all cases. As a retrospective study, treatment details (including ethanol therapy and fluid resuscitation) were not consistently documented for all hospital-presenting cases, which may contribute to variability in measured methanol/ethanol concentrations. Formate, the principal toxic metabolite of methanol, is not routinely measured; therefore, a direct assessment of the metabolic stage was not possible. Furthermore, the study focused exclusively on fatal cases, and direct comparisons between the concentrations in survivors and decedents was not possible. Nevertheless, within these constraints, the combination of robust method validation, multi-matrix analysis, and a larger autopsy cohort than previously reported offers a substantial improvement over existing national data.

## 5. Conclusions

Taken together, the current findings highlight methanol intoxication as an important and preventable cause of death in Jeddah, primarily affecting young and middle-aged adults and occurring in a variety of domestic, occupational, and public settings. This study demonstrated that a meaningful interpretation of methanol concentrations is possible using a multi-matrix approach even in decomposed bodies and supported the utility of validated quantitative methods for defensible forensic conclusions. Multiple matrices were evaluated; however, tissue (mg/g) and fluid (mg/dL) values are not directly comparable, so cross-matrix results are used for complementary/confirmatory interpretation—especially in decomposed cases—rather than dose-equivalent comparisons.

Given the clustering of fatalities into distinct outbreak periods, our findings support strengthening surveillance, early warning, and targeted prevention efforts. Because meaningful interpretation remained possible using multiple matrices even in decomposed bodies, we recommend systematic collection of vitreous humor and key tissues and standardized multi-matrix sampling protocols, particularly when blood may be compromised. To ensure defensible forensic conclusions, we recommend standardized, validated quantitative HS–GC methods for methanol determination. Finally, because methanol intoxication is time-critical and potentially preventable, we recommend rapid, 24/7 access to validated chromatographic methanol testing—ideally within or directly supported by emergency departments—while noting that specific regulatory actions to reduce access to high-risk methanol-containing products require complementary epidemiologic and regulatory data beyond this case series.

## Figures and Tables

**Table 1:** Demographic, case, and specimen (available matrices) characteristics of methanol-related fatalities (Jeddah, 2018–2022; *n* = 34).

Characteristic	Category	*n* (%) or Summary
Number of cases	-	34
Age (years)	Continuous	Mean, 36.9 ± 13.8; median, 34.5; range, 18–73
Age group (years)	<20	1 (2.9%)
	20–29	10 (29.4%)
	30–39	10 (29.4%)
	40–49	8 (23.5%)
	50–59	2 (5.9%)
	60–69	1 (2.9%)
	>70	2 (5.9%)
Sex	Male	28 (82.4%)
	Female	6 (17.6%)
PMI	Continuous (days)	mean, 2.65 ± 2.84; median, 2; range, 1–15
Manner of death	Accidental	24 (70.6%)
	Suicidal	1 (2.9%)
	Unrelated to methanol	3 (8.8%)
	Unknown/undetermined	6 (17.6%)
Location of death	Home	18 (52.9%)
	Hospital	7 (20.6%)
	Outdoor/public place	6 (17.6%)
	Workplace	1 (2.9%)
	Vehicle	1 (2.9%)
	Hotel	1 (2.9%)
Putrefaction at autopsy	None	23 (67.6%)
	Some	7 (20.6%)
	Heavy	4 (11.8%)
Post-mortem samples collected at autopsy	Peripheral blood	12 (35.3%)
	NaF-preserved blood	26 (76.5%)
	Urine	16 (47.1%)
	Vitreous humor	22 (64.7%)
	Gastric contents	7 (20.6%)
	Bile	6 (17.6%)
	Liver	9 (26.5%)
	Kidneys	8 (23.5%)
	Stomach wall	4 (11.8%)
	Bladder tissue	4 (11.8%)
	Small intestine	2 (5.9%)
	Large intestine	1 (2.9%)
	Brain	2 (5.9%)
	Spleen	1 (2.9%)
	Skeletal muscle	1 (2.9%)

**Table 2:** Mann–Whitney U test of outbreak years (2018 vs. 2022).

Endpoint	2018 (*n*) Median [IQR]	2022 (*n*) Median [IQR]	U	*p* (Two-Tailed)	*p* (Holm)	*r_rβ_* (2018 > 2022+)
Methanol concentration in NaF-preserved blood (mg/dL)	10: 281.0 [63.2–441.5]	11: 65.0 [30.5–124.0]	80.0	0.084	0.220	+0.455
Methanol concentration in urine (mg/dL)	7: 456.0 [240.5–514.0]	5: 122.0 [55.0–280.0]	29.0	0.073	0.220	+0.657
Methanol concentration in vitreous humor (mg/dL)	8: 335.0 [61.8–487.2]	10: 61.5 [34.0–111.2]	60.0	0.083	0.220	+0.500
Age (years)	13: 45.0 [26.0–46.0]	12: 33.0 [29.5–35.8]	88.0	0.604	0.604	+0.128
PMI (days)	13: 2.0 [2.0–2.0]	12: 1.0 [1.0–2.0]	114.5	0.036	0.072	+0.468

**Table 3:** Quantitative methanol concentrations in biofluids and tissues in methanol-related fatalities (Jeddah, 2018–2022).

Matrix	*n*	Median (Range)	Mean ± SD
Biofluids			
NaF-preserved blood (mg/dL)	26	101 (10–578)	169.9 ± 167.3
Peripheral blood (mg/dL)	12	244 (11–610)	259.1 ± 205.3
Urine (mg/dL)	15	310 (54–551)	304.1 ± 180.3
Vitreous humor (mg/dL)	22	90 (10–539)	180.0 ± 178.9
Gastric contents (mg/dL)	6	135.5 (1.1–398)	158.0 ± 169.9
Bile (mg/dL)	5	15 (3.6–100)	32.5 ± 39.2
Bladder tissues (mg/g)	3	1.0 (0.72–10.0)	3.9 ± 5.3
Tissues			
Liver (mg/g)	8	1.7 (0.02–99.0)	16.1 ± 34.1
Kidneys (mg/g)	6	2.1 (0.82–95.0)	19.7 ± 37.4
Stomach wall (mg/g)	4	0.9 (0.38–1.4)	0.9 ± 0.4
Brain (mg/g)	2	109.5 (17–202)	109.5 ± 130.8

**Table 4:** Correlation between methanol concentrations and putrefaction (no vs. yes).

Matrix	*n* (Total)	No Putrefaction (*n*)	Putrefaction (*n*)	Mean Concentration–No Putrefaction	Mean Concentration–Putrefaction	Spearman ρ *
NaF-preserved blood	26	21	5	142.8 mg/dL	283.8 mg/dL	0.34
Vitreous humor	22	17	5	179.6 mg/dL	181.4 mg/dL	0.00
Urine	15	11	4	305.4 mg/dL	300.8 mg/dL	−0.01
Liver	8	4	4	5.1 mg/g	27.2 mg/g	0.35

* Spearman ρ: The correlation between putrefaction status and methanol concentration for each matrix.

**Table 5:** Mode of death (toxicological context) and methanol concentrations by matrix.

Mode of Death (Toxicological Context)	Cases (*n*)	%	BNaF (mg/dL)	Blood (mg/dL)	Urine (mg/dL)	Vitreous (mg/dL)	Bile (mg/dL)	Gastric (mg/dL)	Liver (mg/g)	Kidneys (mg/g)
Methanol only	21	61.8	191.0 (34.0–578.0); *n* = 17	318.0 (41.0–610.0); *n* = 9	310.0 (55.0–551.0); *n* = 13	211.0 (14.0–539.0); *n* = 17	100.0 (100.0–100.0); *n* = 1	254.0 (1.1–398.0); *n* = 3	0.6 (0.2–1.1); *n* = 2	1.2 (1.2–1.2); *n* = 1
Methanol + ethanol	5	14.7	53.0 (15.0–91.0); *n* = 2	13.0 (13.0–13.0); *n* = 1	-	25.0 (25.0–25.0); *n* = 1	15.0 (3.6–33.0); *n* = 3	139.0 (12.0–266.0); *n* = 2	4.3 (1.1–19.0); *n* = 4	2.1 (0.8–17.0); *n* = 4
Unrelated/low-level methanol	4	11.8	11.0 (10.0–15.0); *n* = 4	11.0 (11.0–11.0); *n* = 1	-	14.0 (10.0–18.0); *n* = 2	11.0 (11.0–11.0); *n* = 1	17.0 (17.0–17.0); *n* = 1	0.0 (0.0–0.0); *n* = 1	-
Methanol + other drugs (no ethanol)	3	8.8	59.0 (27.0–270.0); *n* = 3	86.0 (86.0–86.0); *n* = 1	222.5 (54.0–391.0); *n* = 2	54.5 (29.0–80.0); *n* = 2	-	-	-	-
Methanol + ethanol + other drugs	1	2.9	-	-	-	-	-	-	99.0 (99.0–99.0); *n* = 1	95.0 (95.0–95.0); *n* = 1

BNaF, NaF-preserved blood.

**Table 6:** Manner of death and methanol concentrations across biological matrices.

Manner of Death	Cases (*n*)	NaF-Preserved Blood (mg/dL)	Peripheral Blood (mg/dL)	Urine (mg/dL)	Vitreous Humor (mg/dL)	Bile (mg/dL)	Liver (mg/g)	Kidneys (mg/g)
Accidental	24	138 (10–578)	244 (11–610)	310 (54–551)	107 (10–539)	185 (42–720)	1.7 (0.17–7.2)	2.1 (0.3–6.4)
Undetermined	6	75 (28–310)	110 (34–402)	190 (65–480)	90 (18–475)	160 (55–510)	1.2 (0.3–4.1)	1.8 (0.4–3.9)
Suicide	1	412	-	-	365	-	3.6	4.2

**Table 7:** Seasonal distribution of methanol-related fatalities (*n* = 34).

Season	Months Included	Number of Cases (*n*)	Percentage (%)
Winter	December–February	6	17.6
Spring	March–May	6	17.6
Summer	June–August	7	20.6
Autumn	September–November	15	44.1
Total	-	34	100.0

**Table 8:** Spearman’s correlation between methanol concentrations in different matrices.

Matrix	Blood	NaF-Preserved Blood	Urine	Vitreous Humor	Gastric Contents
Blood	1.000 (-, *n* = 22)	NA	NA	NA	NA
NaF-preserved blood	NA	1.000 (-, *n* = 6)	NA	−0.316 (*p* = 0.684, *n* = 4)	−0.500 (*p* = 0.667, *n* = 3)
Urine	NA	NA	1.000 (-, *n* = 5)	−0.500 (*p* = 0.667, *n* = 3)	−0.500 (*p* = 0.667, *n* = 3)
Vitreous humor	NA	−0.316 (*p* = 0.684, *n* = 4)	−0.500 (*p* = 0.667, *n* = 3)	1.000 (-, *n* = 8)	0.986 (*p* = 0.0003, *n* = 6)
Gastric	NA	−0.500 (*p* = 0.667, *n* = 3)	−0.500 (*p* = 0.667, *n* = 3)	0.986 (*p* = 0.0003, *n* = 6)	1.000 (2013, *n* = 6)

NA, not applicable.

**Table 9:** Mann–Whitney U test of methanol concentrations (case-level analysis).

Comparison No.	Endpoint	Group 1 (*n*)	Median [IQR]	Group 2 (*n*)	Median [IQR]	U	*p* (Two-Tailed)	*p* (Holm)	*r_rβ_* (Signed)
1	Vitreous methanol (mg/dL)	17	100.0 [49.0–235.0]	5	65.0 [52.0–290.0]	43.0	1.0000	1.0000	+0.0118
2	Urine methanol (mg/dL)	11	310.0 [163.0–435.5]	4	312.5 [146.0–467.2]	22.0	1.0000	1.0000	+0.0000
3	BNaF methanol (mg/dL)	22	144.0 [55.2–288.0]	4	38.0 [22.8–71.2]	67.5	0.1020	0.5101	+0.5341
4	BNaF methanol (mg/dL)—accidental only	10	193.5 [117.0–278.5]	6	321.0 [85.0–486.5]	26.0	0.7128	1.0000	−0.1333
5	Max biofluid methanol (mg/dL)	18	295.0 [99.5–455.5]	5	86.0 [54.0–266.0]	67.0	0.1109	0.5101	+0.4889

BNaF, NaF-preserved blood.

## Data Availability

The original contributions presented in this study are included in the article. Further inquiries can be directed to the corresponding authors.

## Abbreviations

The following abbreviations are used in this manuscript:

ANSI/ASB	American National Standards Institute/ASB Standards Board
COVID-19	Coronavirus disease 2019
CV	Coefficient of variation
FID	Flame ionization detector
GC	Gas chromatography
HS–GC–FID	Headspace gas chromatography–flame ionization detector
LOD	Limit of detection
LOQ	Limit of quantification
MRI	Magnetic resonance imaging
NaF	Sodium fluoride
PMI	Post-mortem interval
QC	Quality control
SD	Standard deviation
SPSS	Statistical Package for the Social Sciences
THC	Δ^9^-tetrahydrocannabinol

## References

[1] Celik S., Karapirli M., Kandemir E., Ucar F., Kantarci M.N., Gurler M., Akyol O. (2013). Fatal Ethyl and Methyl Alcohol-Related Poisoning in Ankara: A Retrospective Analysis of 10,720 Cases Between 2001 and 2011. J. Forensic Leg. Med..

[2] Al-Asmari A.I., Al-Amoudi D.H. (2020). The Role of Ethanol in Fatalities in Jeddah, Saudi Arabia. Forensic Sci. Int..

[3] Alsayed S.N., Alharbi A.G., Alhejaili A.S., Aljukhlub R.J., Al-Amoudi D.H., Ashankyty A.I., Alzahrani M.A., Zughaibi T.A., Alharbi O.A., Kheyami A.M. (2022). Ethyl Glucuronide and Ethyl Sulfate: A Review of Their Roles in Forensic Toxicology Analysis of Alcohol Postmortem. Forensic Toxicol..

[4] Eskandrani R., Almulhim K., Altamimi A., Alhaj A., Alnasser S., Alawi L., Aldweikh E., Alaufi K., Mzahim B. (2022). Methanol Poisoning Outbreak in Saudi Arabia: A Case Series. J. Med. Case Rep..

[5] Kabli A.O., Felemban A.M., Nasri A.K., Aljedani F.A., Bedairi A.M., Alghamdi M.M., Alghamdi A.S., Ogran S.Y. (2023). Outcome of Methanol Toxicity Outbreak In Saudi Arabia: Case Series Study. Cureus.

[6] Alhusain F., Alshalhoub M., Homaid M.B., Esba L.C.A., Alghafees M., Al Deeb M. (2024). Clinical Presentation and Management of Methanol Poisoning Outbreaks in Riyadh, Saudi Arabia: A Retrospective Analysis. BMC Emerg. Med..

[7] Behnoush A.H., Bazmi E., Khalaji A., Jafari-Mehdiabad A., Barzegari N., Dehpour A.R., Behnoush B. (2024). The Trend of Poisonings Before and After the COVID-19 Pandemic. Sci. Rep..

[8] Arasteh P., Pakfetrat M., Roozbeh J. (2020). A Surge in Methanol Poisoning Amid COVID-19 Pandemic: Why Is This Occurring?. Am. J. Med. Sci..

[9] Mousavi-Roknabadi R.S., Arzhangzadeh M., Safaei-Firouzabadi H., Mousavi-Roknabadi R.S., Sharifi M., Fathi N., Zarei Jelyani N., Mokdad M. (2022). Methanol Poisoning During COVID-19 Pandemic; A Systematic Scoping Review. Am. J. Emerg. Med..

[10] Alqurashi G.I., Alqurashi F.S., Alhusayni K.M., Falemban A.H., Alhindi Y.Z., Alsanosi S.M., Alzahrani A.R., Al-Ghamdi S.S., Ayoub N. (2023). Case Reports Study on Methanol Poisoning in King Abdul Aziz Specialist Hospital, Taif, Saudi Arabia. J. Clin. Med..

[11] Tobaiqy M., Al-Asmari A.I. (2024). Substance Misuse Disorder in Saudi Arabia: A Comprehensive Examination of Current Demographic Patterns, Trends, and Intervention Requirements. Saudi Pharm. J..

[12] Alnefaie S.A., Aldlgan A.A., Albakiri K.M., Kaabi M.A., Alzwen G.M., Al-Otaibi S.S., Alasmari F. (2024). Methanol Intoxication in the Central Region of Saudi Arabia: Five Case Studies. Saudi Pharm. J..

[13] Galvez-Ruiz A., Elkhamary S.M., Asghar N., Bosley T.M. (2015). Visual and Neurologic Sequelae of Methanol Poisoning in Saudi Arabia. Saudi Med. J..

[14] Aisa T.M., Ballut O.M. (2016). Methanol Intoxication with Cerebral Hemorrhage. Neurosciences.

[15] (2019). Method Validation in Forensic Toxicology.

[16] Shokoohi M., Nasiri N., Sharifi H., Baral S., Stranges S. (2020). A Syndemic of COVID-19 and Methanol Poisoning in Iran: Time for Iran to Consider Alcohol Use as a Public Health Challenge?. Alcohol.

[17] Holzman S.D., Larsen J., Kaur R., Smelski G., Dudley S., Shirazi F.M. (2021). Death by Hand Sanitizer: Syndemic Methanol Poisoning in the Age of COVID-19. Clin. Toxicol..

[18] Vaibhav V., Shukla P.K., Meshram R., Bhute A.R., Varun A., Durgapal P. (2022). Methanol Poisoning: An Autopsy-Based Study at the Tertiary Care Center of Uttarakhand, India. Cureus.

[19] Chng K.L., Lai P.S., Siew S.F., Md Yaro S.W., Mahmood M.S. (2020). Methanol Related Death in National Institute of Forensic Medicine, Hospital Kuala Lumpur: A Case Series. Malays. J. Pathol..

[20] Alasmari A., Alhejaili A., Alharbi H., Alzahrani M., Zughaibi T. (2024). Challenges and Insights: Methamphetamine Analysis in Post-Mortem Putrefied Human Tissues in a Hot Climate. Saudi Pharm. J..

[21] Al-Asmari A.I., Altowairgi M.M., Al-Amoudi D.H. (2022). Effects of Post-mortem Interval, Putrefaction, Diabetes, and Location of Death on the Analysis of Ethyl Glucuronide and Ethyl Sulfate as Ethanol Biomarkers of Antemortem Alcohol Consumption. Forensic Sci. Int..

[22] Beno J.M., Hartman R., Wallace C., Nemeth D., LaPoint S. (2011). Homicidal Methanol Poisoning in a Child. J. Anal. Toxicol..

[23] Tomsia M., Głaz M., Nowicka J., Cieśla J., Sosnowski M., Chełmecka E. (2022). Fatal Methanol Poisoning Caused by Drinking Industrial Alcohol: Silesia Region, Poland, April–June 2022. Toxics.

[24] Wu Chen N., Donoghue E., Schaffer M. (1985). Methanol Intoxication: Distribution in Postmortem Tissues and Fluids Including Vitreous Humor. J. Forensic Sci..

